# A Role for Ethanol-Induced Oxidative Stress in Controlling Lineage Commitment of Mesenchymal Stromal Cells Through Inhibition of Wnt/β-Catenin Signaling

**DOI:** 10.1002/jbmr.7

**Published:** 2009-12-21

**Authors:** Jin-Ran Chen, Oxana P Lazarenko, Kartik Shankar, Michael L Blackburn, Thomas M Badger, Martin J Ronis

**Affiliations:** 1 Department of Pediatrics Little Rock, AR, USA; 2 Department of Physiology and Biophysics Little Rock, AR, USA; 3 Department of Pharmacology and Toxicology, University of Arkansas for Medical Sciences Little Rock, AR, USA; 4 Arkansas Children's Nutrition Center Little Rock, AR, USA

**Keywords:** ethanol, wnt, β-catenin, osteoblast, *N*-acetylcysteine

## Abstract

The mechanisms by which chronic ethanol intake induces bone loss remain unclear. In females, the skeletal response to ethanol varies depending on physiologic status (e.g., cycling, pregnancy, or lactation). Ethanol-induced oxidative stress appears to be a key event leading to skeletal toxicity. In this study, ethanol-containing liquid diets were fed to postlactational female Sprague-Dawley rats intragastrically for 4 weeks beginning at weaning. Ethanol consumption decreased bone mineral density (BMD) compared with control animals during this period of bone rebuilding following the end of lactation. Coadministration of the antioxidant *N*-acetylcysteine (NAC) was able to block bone loss and downregulation of the bone-formation markers alkaline phosphatase and osteocalcin in serum and gene expression in bone. Real-time array analysis of total RNA isolated from bone tissue revealed that the majority of Wnt signaling components were downregulated by chronic ethanol infusion. Real-time PCR confirmed downregulated gene expression in a subset of the Wnt signaling components by ethanol. However, the Wnt antagonist DKK1 was upregulated by ethanol. The key canonical Wnt signaling molecule β-catenin protein expression was inhibited, while glycogen synthase kinase-3-β was dephosphorylated by ethanol in bone and preosteoblastic cells. These actions of ethanol were blocked by NAC. Ethanol treatment inactivated *TCF/LEF* gene transcription, eliminated β-catenin nuclear translocation in osteoblasts, and reciprocally suppressed osteoblastogenesis and enhanced adipogenesis. These effects of ethanol on lineage commitment of mesenchymal stem cells were eliminated by NAC pretreatment. These observations are consistent with the hypothesis that ethanol inhibits bone formation through stimulation of oxidative stress to suppress Wnt signaling. © 2010 American Society for Bone and Mineral Research.

## Introduction

Substantial changes in maternal skeletal metabolism occur to provide the minerals required for development of the fetal and neonatal skeleton during pregnancy and lactation.(1) Observations from both human and animal studies have revealed that remarkable bone loss often accompanies lactation, mainly owing to calcium stress.(2,3) Subsequently, rapid and substantial gains in bone mineral content and density occur in the maternal skeleton immediately after lactation ends. Previous evidence from studies in rodents show that during the first 48 hours after weaning, rapid inactivation and apoptosis of osteoclasts is the trigger for a profoundly anabolic period in the dam characterized by greatly increased bone formation.(4,5) This postlactational bone-formation period in the maternal skeleton is accompanied by greatly increased proliferation of osteoblast progenitors resulting in a rapid expansion of osteoblasts on bone surfaces in the maternal skeleton.(4)

Osteoblast differentiation is driven by numerous factors. The Wnt/β-catenin signaling pathway is a potent regulator responsible for directing mesenchymal stem cells to differentiate toward the osteoblastic lineage, resulting in more bone formation and less adipogenesis.(6) Wnt activates a receptor complex made by Frizzled and the low-density lipoprotein–related proteins 5 or 6 (LRP5 or LRP6), leading to inactivation of glycogen synthase kinase 3β (GSK-3β). This prevents proteosomal degradation of the transcriptional coactivator β-catenin and thereby promotes its accumulation in the cytoplasm.(7,8) β-Catenin translocates into the nucleus, where it associates with the T cell factor (TCF) lymphoid-enhancer binding factor (LEF) family of transcription factors and regulates the expression of Wnt target genes.(9)

Both chronic and binge ethanol consumption causes suppression of bone formation and increased bone resorption, and alcohol is considered as a major risk factor for osteoporosis, a disease particularly prevalent in women worldwide.(10,11) Moreover, bone remodeling differs remarkably during specific physiologic states in women, such as pregnancy, lactation, and menopause, owing to profound changes in the systemic endocrine and mineral milieu during those periods.(12) Thus alcohol abuse may disrupt bone turnover during these particular physiologic periods differently than during other periods, such as menstrual cycling. Few studies have been conducted on the skeletal effects of alcohol consumption in pregnant or lactating women, and results from postmenopausal women are inclusive, most likely because of differing amounts and duration of alcohol intake.(13)

We recently reported that ethanol (EtOH) dramatically decreased bone mineral density (BMD) in nonpregnant cycling female rats, whereas pregnant animals had less bone loss in response to identical doses of EtOH.(14) We also reported that postlactating rats showed suppression of the rapid bone-rebuilding phase after weaning as early as 1 week after EtOH intake.(15) Although the physiologic changes in bone of postlactating animals are themselves poorly understood, exploring the mechanism of alcohol effects on bone during this period may be important because large numbers of women who abstain from alcohol during pregnancy and lactation on the advice of physicians resume drinking after the end of breast-feeding. We have previously hypothesized that if women continue to drink during this postlactational period, the rapid bone-rebuilding phase will be disrupted, normal bone density will never be restored, and consequently, greater risk of bone fracture would be expected later in life.(16)

We previously hypothesized that oxidative stress is the pivotal pathogenetic factor mediating chronic alcohol-related bone loss and strength in rats.(17) Oxidative stress leads to a decrease in the rate of bone formation and an acceleration of osteoblast senescence.(18) Recent evidence also has suggested a linkage between oxidative stress, Wnt/β-catenin signaling, osteoblastogenesis, adipogenesis, osteoporosis, and atherosclerosis.(19) Wnt signaling has been suggested to be a potential target for EtOH,(11) but the molecular mechanisms remain obscure. In this study we demonstrate that downregulation of canonical Wnt/β-catenin signaling is associated with suppression of anabolic bone rebuilding by chronic alcohol consumption postlactation and that the dietary antioxidant *N*-acetylcysteine (NAC) blocks ethanol effects on Wnt signaling in vivo. We also provide evidence that alcohol eliminates β-catenin nuclear translocation and targeted gene transcription in osteoblast pecursors in vitro. Our data indicate that ethanol influences the lineage commitment of mesenchymal stem cells in bone marrow.

## Materials and Methods

### Animal experiments

Female pregnant Sprague-Dawley rats (250 to 300 g) were purchased from Charles River Laboratories, Inc. (Wilmington, MA, USA) on gestation day 12. Animals were housed in an Association for the Assessment and Accreditation of Laboratory Animal Care–approved animal facility. Animal maintenance and experimental treatments were conducted in accordance with the ethical guidelines for animal research established and approved by the Institutional Animal Care and Use Committee at the University of Arkansas for Medical Science (Little Rock, AR, USA). Rats were surgically implanted with an intragastric cannula and infused with water (25 mL/day) as described previously.(20,21) The rats had access to commercial rodent diet and water *ad libitum* during the rest of gestation and lactation. At birth, litters were culled to 5 male and 5 female pups per dam, and litter weights were equalized. Rat dams (*n* = 8/group) were randomly assigned to four groups immediately after weaning (on postnatal day 17) and fed by total enteral nutrition (TEN). Liquid diets were formulated to contain the nutrients recommended for rats by the National Research Council. The TEN animal model has been detailed previously.(22) Two groups of dams received control diets at 220 kcal/kg^3/4^ per day with or without the antioxidant *N*-acetyl cysteine (NAC, 1.4 g/kg/day) as a part of their liquid diets. The other two groups of rats received EtOH-containing diets (12 g/kg per day) with or without NAC (1.4 g/kg/day). Diets contained 16% protein, 54% carbohydrate, and 25% fat (corn oil), and EtOH-containing diets were kept isocaloric to the control diets by substituting EtOH for carbohydrate calories. Rats were infused for 14 hours from 6:00 p.m. to 8:00 a.m. during the dark cycle for 4 weeks. At the completion of the experiment, rats were killed under anesthesia, and serum, left tibiae, and left femora were collected. Formalin-fixed left tibial peripheral quantitative computed tomography (pQCT) was performed for BMD measurement using a method well established previously in our laboratory.(14,22) All rats were weighed every other day, and we found that there were no significant differences among all four groups in body weight at the end of experiment.

### Serum bone-formation marker ALP measurement

The serum bone-formation marker bone-specific alkaline phosphatase (ALP) was measured by a colorimetric assay using the time-dependent formation of *p*-nitrophenolate from *p*-nitrophenyl phosphate (PNPP).(23) The protocol used 5 µL of serum in a total volume of 1.5 mL containing 0.05 mmol/L PNPP, 2 mmol/L MgCl_2_, and 10 mmol/L l-phenylalanine (to inhibit any circulating intestinal ALP activity).

### RNA isolation and Wnt real-time PCR array

Rat bone marrow cells from all four groups were harvested from femora according to methods described previously.(24) Bone marrow and bone marrow–flushed femur bone RNAs were extracted using TRI Reagent (MRC, Inc., Cincinnati, OH, USA) according to the manufacturer's recommendations followed by DNase digestion and column cleanup using RNeasy minicolumns (Qiagen, Valencia, CA, USA) as described previously.(14) Wnt signaling pathway–focused gene expression array analysis was performed using a kit of RT^2^ Profiler PCR Array System purchased from SuperArray Bioscience Corporation (Frederick, MD, USA). The procedure followed instructions provided by the manufacturer. Reverse transcription was carried out using an iScript cDNA synthesis kit from Bio-Rad (Hercules, CA, USA). RNA isolation from in vitro cell culture also was described previously.(18) Validation of subset gene array data and gene expression in bone tissues and cells from in vitro studies was carried out by real-time RT-PCR using SYBR Green and an ABI 7000 sequence detection system (Applied Biosystems, Foster City, CA, USA). All primers for real-time PCR analysis were designed using Primer Express Software 2.0 (Applied Biosystems) and are listed in Supplemental Table 1.

### Cell cultures

In order to obtain stromal osteoblasts, bone marrow cells were seeded at a density of 3 × 10^6^ and 1.5 × 10^6^ cells per well in 6- and 24-well cell culture plates in the presence of minimum essential medium (MEM) (Invitrogen, Carlsbad, CA, USA) with 10% fetal bovine serum (FBS, Hyclone Laboratories, Logan, UT, USA) and 1 mM of ascorbyl-2-phosphate (Sigma-Aldrich, St. Louis, MO, USA), 4 mM L-glutamine and 100 U/mL each of penicillin and streptomycin (Sigma-Aldrich), conditions known to drive osteoblast differentiation. Half the osteoblast differentiation medium (OB medium) was changed every 2 days, and *N*-acetyl cysteine (NAC) 1 mM and/or ethanol (EtOH) 50 mM were added into culture medium. Culture plates were double sealed with transparent tape and parafilm. Since this was long-term cell culture with EtOH, the culture medium was saturated with oxygen and CO_2_ in the incubator for 4 hours before the changing of the culture medium. After 22 days, mature osteoblasts were developed for von Kossa staining and RNA collection. Neonatal rat calvarial osteoblastic cells were isolated from untreated 4-day-old rat pups by sequential collagenase digestion using a method described previously.(25) Rat calvarial osteoblastic cells were cultured and treated with NAC and EtOH in the same fashion as the bone marrow cells described earlier with or without the presence of 1 mM of ascorbyl-2-phosphate. Calvarial cells were cultured for 10 days in 24-well plates for ALP staining. Bone marrow stromal cell line ST2 cells were cultured in α-MEM supplemented with 10% FBS in the presence or absence of 50 ng/mL of soluble Wnt 3a (R&D Systems, Inc., Minneapolis, MN, USA). ST2 cells were treated with or without NAC, EtOH, or their combination in a similar fashion to the treatment of bone marrow cells ex vivo in the 24- and 6-well culture plates for 7 days for ALP staining and collection of protein for Western blotting. To assess adipogenesis, C3H10T1/2 cells were cultured in DMEM supplemented with 10% FBS and treated similarly to bone marrow cells with NAC, EtOH, and their combination. Cells were supplemented with methyl-isobutylxanthine (0.5 mM), dexamethasone (0.25 µm), and insulin (10 µg/mL) 2 days after confluence. Forty-eight hours later, this medium was withdrawn and replaced with medium supplemented only with insulin (10 µg/mL). Oil red O staining in 24-well plates and RNA collection from 6-well plates were performed.

### DNA constructs, luciferase activity assays, transient transfection, and subcellular localization of β-catenin-mediated signaling

A β-catenin Green Fluorescent Protein (GFP) construct was generated by inserting a full-length rat β-catenin 2.3-kb PCR product into a pEGFP-N1 vector (Clontech, Mountain View, CA, USA). Briefly, RNA isolated from rat calvarial cells was used for reverse transcription using an iScript cDNA synthesis kit from Bio-Rad. High-fidelity PCR amplication (PFX kit from Invitrogen) of β-catenin was obtained using forward primer 5'-CAGGAGCTCTGGACAATGGCTACTCAAGCTGACC-3' and reverse primer 5'-ACGGGATCCAGGTCGGTATCAAACCAGGCCA-3'. PCR products were digested with *Sac*I and *Bam*HI and inserted into the same sites in the appropriate pEGFP-N1 vector. TCF/LEF-Firefly luciferase reporter plasmid (TOPFLASH) and control reporter containing mutant TCF biding sites (FOPFLASH) were purchased from Upstate Biotechnology, Lake Placid, NY, USA. Using 24-well plates, rat osteoblastic UMR-106 cells (ATCC, Rockville, MD, USA) were transiently transfected with 0.005 µg of TOPFLASH or 0.005 µg of FOPFLASH plasmid and 0.025 µg of empty pEGFP-N1 vector. Constitutively active pRL-CMV Renilla luciferase vector (0.005 µg, Promega) was used as an internal control for transfection efficiency. Following transfection, cells were allowed to grow overnight before being treated with NAC, EtOH, and their combination at the same concentrations used in previous bone marrow cells. Then, 24 hours after cell treatment, Renilla luciferase activity was determined using the dual luciferase assay system according to the manufacturer's instructions (Promega, Madison, WI, USA). Luciferase activity was measured on an MLX Microtiter Plate Luminometer (Dynex Technolnogies, Inc., Chantilly, VA, USA). For the β-catenin nuclear translocation experiment, full-length wild-type β-catenin-GFP plasmid along with red fluorescent protein (pDs 1Red-N1, Clontech, Palo Alto, CA, USA) targeted to the nucleus (nRFP)(26,27) was transiently cotransfected into UMR-106 cells in 24-well plates using Lipofectamine 2000 (Invitrogen). Transfected cells were cultured for 24 hours. Subsequently, cells were serum-starved by culturing in the presence of 2% bovine serum albumin (BSA) for 4 hours and treated with vehicle, 50 mM EtOH, 1 mM NAC, and their combination for 6 hour. Cells showing either nuclear or cytoplasmic accumulation of β-catenin were visualized directly using a fluorescence microscope.

### Western blotting and protein carbonylation assays

Tibia bone tissue proteins and in vitro cellular proteins were extracted using a cell lysate buffer as described previously.(17) β-Catenin, phosphorylation of GSK-3β, and total GSK-3β and β-actin in bone tissue and in vitro osteoblasts were assessed by Western immunoblotting using goat polyclonal antibody recognizing β-catenin (Cell Signaling, Danvers, MA, USA), rabbit polyclonal antibodies recognizing phosphorylated GSK-3β and total GSK-3β (Cell Signaling), and mouse polyclonal antibody recognizing β-actin (Sigma), followed by incubation with either an anti-goat, an anti-rabbit, or an anti-mouse secondary antibody conjugated with horseradish peroxidase (Santa Cruz Biotechnology, Santa Cruz, CA, USA). SuperSignal West Pico chemiluminescent substrate (Pierce, Rockford, IL, USA) was used for developing blots. For the protein carbonylation assay, carbonyl levels were determined in 10 µg of ST2 cell protein lysate using a commercial kit (OxyBlot, Serologicals, Norcross, GA, USA) according to the manufacturer's instructions. Quantitation of the intensity of the bands in the autoradiograms was performed using a VersaDoc imaging system (Bio-Rad, Hercules, CA, USA).

### Statistical analysis

Data were expressed as means ± SD. One- and two-way analyses of variance (ANOVA) followed by Student-Newman-Keuls post hoc analysis were used to compare the treatment groups. Values were considered statistically significant at *p* < .05.

## Results

### EtOH-induced reduction in BMD is associated with reduced bone formation in female rats after lactation

In the EtOH-infused (13 g/kg/day) group, trabecular BMD was lower compared with the control group (control 231.8 ± 12 mg/cm^3^ versus EtOH 172.6 ± 9.2 mg/cm^3^, *n* = 7, *p* < .05). NAC supplementation of the EtOH diet attenuated the effects on tibial trabecular BMD (223.8 ± 8.5 mg/cm^3^, *n* = 7, *p* < .05 versus EtOH) while not differing from NAC controls (224.0 ± 11.5 mg/cm^3^, *n* = 7). These results were similar to those we previously published.(15) Since postlactational bone rebuilding depends mainly on substantial osteoblastic bone formation, we measured bone-formation markers in rat serum and their gene expression in bone tissue. A lower level of bone-specific alkaline phosphatase (ALP) activity was found in the EtOH-treated group compared with the control group (1*A*) (*p* < .05). These effects on bone turnover markers are consistent with the reduced bone-formation rates observed previously in our laboratory in EtOH-treated postlactating dams by dynamic histomorphometry.(15) Sera from animals treated with NAC alone or the combination of NAC and EtOH had similar ALP activity to controls (1*A*). Using RNA extracted from tibial bone, real-time PCR was carried out for two bone-formation genes, *ALP* and *osteocalcin*. In the EtOH-treated group, downregulation of both *ALP* and *osteocalcin* gene expression (*p* < .05) was observed. Administration of NAC attenuated EtOH effects on both *ALP* and *osteocalcin* gene expression (1*B*).

**Fig. 1:**
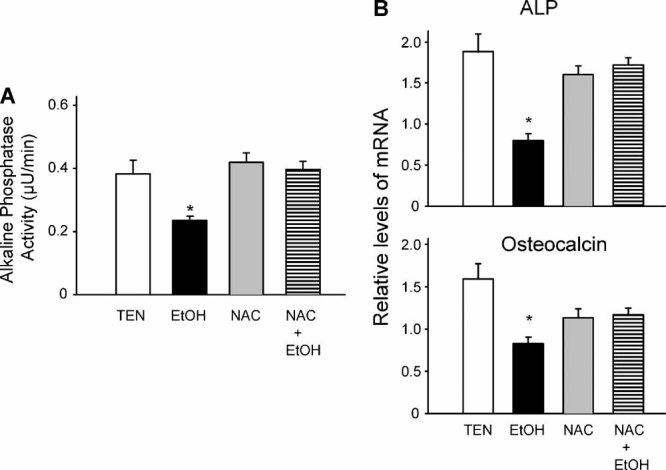
Effect of EtOH treatment on bone-formation markers in female rats 4 weeks after lactation. (*A*) Serum bone-specific ALP activity. (*B*) *ALP* and *osteocalcin* mRNA expression in RNA extracted from rat tibial bone. Data are expressed as mean ± SD (*n* = 7/group). *p < .05 versus TEN control group by ANOVA followed by Student-Newman-Keuls post hoc analysis for multiple pairwise comparisons.

### NAC blocks chronic EtOH infusion–induced suppression of Wnt signaling in bone in postlactational female rats

**Fig. 2:**
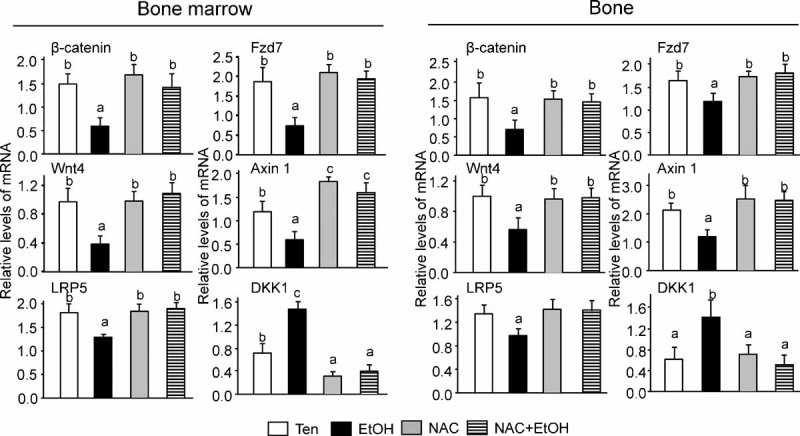
EtOH regulates gene expression of selected Wnt signaling components. Real-time PCR analysis of *β-Catenin*, *Axin1*, *Wnt4*, *LRP5*, *Fzd7*, and *DKK1* in RNA isolated from either bone marrow or bone tissue after aspiration of bone marrow cells. Data are expressed as mean ± SD (*n* = 7/group). Means with different letters differ significantly from each other *p* < .05, *a* < *b* < *c*, as determined by one-way ANOVA followed by Student-Newman-Keuls post hoc analysis for multiple pairwise comparisons.

**Fig. 3:**
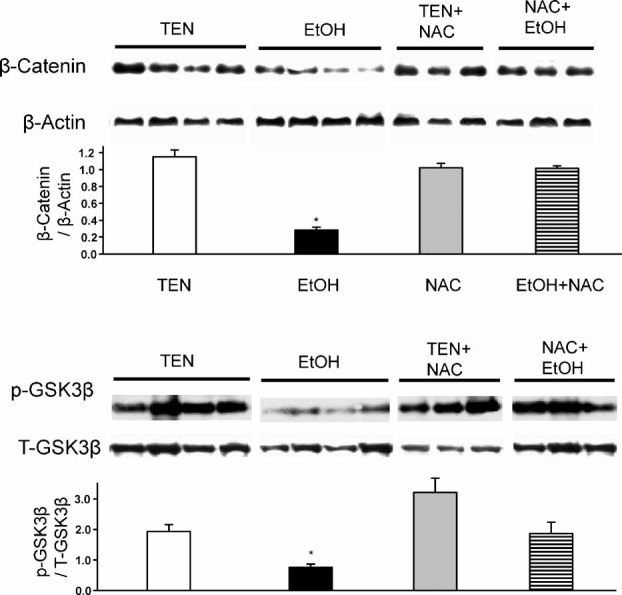
EtOH downregulates *β-catenin* expression in bone. *β-catenin* and *GSK3β* phosphorylation in tibiae assessed by Western blot analysis. The bars represent the ratio of intensity of the band of *β-catenin* over β-actin and phosphorylated *GSK3β* over total GSK-3β protein for each four samples from TEN and EtOH and each three samples from NAC and NAC + EtOH groups using Quantity One Software (Bio-Rad). Data are expressed as mean ± SD. *p < .05 versus TEN control group by ANOVA followed by Student-Newman-Keuls post hoc analysis for multiple pairwise comparisons.

RT^2^ Profiler PCR arrays were used to probe the Wnt signaling pathway–focused gene expression profile associated with the different treatments. Wnt signaling–related genes are listed in Supplemental Table 2. Data were normalized with housekeeping gene *GAPDH* and analyzed based on a ΔΔ*C*_*t*_ method with an Excel-based PCR array data template form provided by the manufacturer. From a total 89 genes, we found that there are 36 genes that have threefold changes and *p* values of less than .05 between control TEN and EtOH groups. Among those 36 genes, there are 12 genes that have threefold changes and *p* values of less than .05 between the EtOH and EtOH + NAC groups. These 12 genes are listed in Table 1. We found that the levels of *β-catenin*, *Wnt4*, and *LRP5*, which are commonly considered as Wnt signaling–positive regulators, were lower, whereas the levels of *DKK1*, which is usually considered a Wnt signaling antagonist, was higher in the EtOH-treated animals compared with TEN diet controls. NAC supplementation blocked the effects of EtOH on both positive regulators and antagonists of Wnt signaling. Interestingly, NAC itself also changed the levels of some Wnt signaling–positive regulators and antagonists compared with TEN controls. A subset of Wnt signaling–associated positive regulators and antagonists was confirmed by real-time PCR (Fig. 2) using RNA isolated from both bone marrow and bone marrow–free bone tissue. In RNA isolated from bone marrow, *β-catenin*, *Axin1*, *Wnt4*, *LRP5*, and *Fzd7* mRNAs were significantly downregulated, whereas *DKK1* was upregulated by EtOH infusion. Consistent with gene array data, NAC antagonized these effects of EtOH on gene expression, and NAC itself significantly upregulated *Axin1* gene expression but downregulated *DKK1* gene expression compared with TEN controls (Fig. 2). Similarly, in RNA isolated from bone tissue, *β-catenin*, *Axin1*, *Wnt4*, *LRP5*, and *Fzd7* mRNAs were significantly downregulated, whereas *DKK1* was upregulated by EtOH infusion. However, we did not find any effect of NAC itself (Fig. 2). This implies that there are other cell types in bone marrow compared with mineralized bone that are more sensitive to the actions of NAC on Wnt signaling. EtOH treatment decreased *β-catenin* mRNA in bone marrow, and this was associated with decreased protein expression (Fig. 3). GSK-3β, which is tightly linked to β-catenin cytoplasmic degradation, also was dephosphorylated and thus activated by EtOH treatment (Fig. 3). These data indicate that EtOH not only may inhibit β-catenin transcription but also may promotes β-catenin cytoplasmic degradation through dephosphorylation of GSK-3β. NAC supplementation attenuated these effects of EtOH on β-catenin protein expression and GSK-3β dephosphorylation (Fig. 3).

**Table 1:** Wnt Signaling Genes Significantly Regulated by EtOH but Reversed by NAC

	EtOH vs. TEN		EtOH + NAC vs. EtOH		NAC vs. TEN	
			
Wnt signaling genes	Fold changes	*p* Value	Fold changes	*p* Value	Fold changes	*p* Value
*Axin1*	−6.98	.0301	13.08	.0464	8.89	.0317
*Ctnnb1*	−75.70	.0115	81.51	.0008	−1.14	.9565
*Ccnd1*	−22.14	.0443	17.70	.0013	4.23	.1257
*Csnk2a1*	−3.31	.0388	9.27	.0139	3.82	.0882
*Dkk1_predicted*	3.14	.0482	−17.08	.0105	−6.44	.0259
*Fzd7_predicted*	−21.84	.0216	5.07	.0437	1.15	.9226
*Gsk3b*	−3.02	.0515	12.54	.0176	4.72	.0825
*Lrp5_predicted*	−6.37	.0330	8.22	.0213	2.64	.3826
*Ppp2r1a*	−4.40	.0415	14.48	.0177	4.97	.0789
*Wnt2*	−5.29	.0178	6.33	.0499	2.51	.2881
*Wnt2b*	−7.09	.0149	4.17	.0455	1.42	.6727
*Wnt4*	−10.24	.0131	6.21	.0241	2.23	.4541

*Note:* This table represents real-time PCR array data. RNA isolated from bone marrow cells with three samples per group. A list of 12 genes was selected from a total of 89 genes identified as affiliated with the Wnt signaling pathway by the PANTHER classification system and by criteria of which showed threefold changes and *p* values of less than .05 between TEN control versus EtOH and between EtOH versus EtOH + NAC. The changes of those 12 genes between TEN and NAC also were presented in the last two columns. Wnt signaling antagonist is highlighted. Data were normalized with housekeeping gene *GAPDH* and statistical comparisons analyzed based on a ΔΔ*C*_*t*_ method with an Excel-based PCR array data template form provided by the manufacturer. Minus symbol = downregulated. The individual full gene names are cited in ref. (11).

### NAC antagonizes EtOH's inhibitory effect on TCF/LEF-dependent transcription and β-catenin nuclear translocation in osteoblasts in vitro

We next examined the ability of EtOH to effect TCF/LEF-dependent transcription in osteoblasts in vitro by cotransfecting cells with full-length *β-catenin-EGFP* fusion constructs and a *TOPFLASH TCF/LEF* reporter plasmid. Transfected cells were treated with EtOH, NAC, and their combination for 24 hours. Treatment with EtOH potently suppressed *TOPFLASH* reporter gene transcription in osteoblastic cells compared with cells treated with vehicle (4*A*). NAC itself did not affect *TOPFLASH* reporter gene transcription, but it completely blocked EtOH's effect (4*A*). Fluorescent microscopy revealed that nuclear translocated *β-catenin-EGFP* was degraded after exposure to 50 mM EtOH (4*B*). On the other hand, in the presence of NAC, nuclear *β-catenin-EGFP* remained in the nucleus following EtOH treatment (4*B*).

**Fig. 4:**
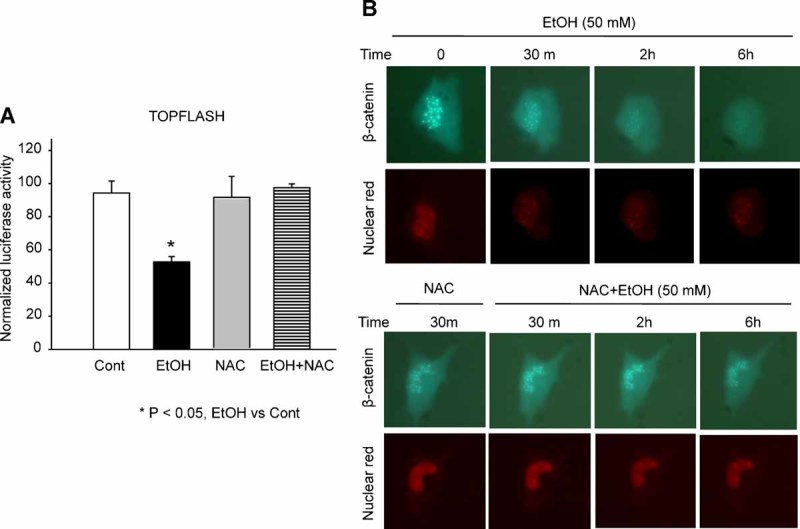
EtOH transinactivates *TCF/LEF* gene transcription in osteoblasts. (*A*) EtOH 50 mM significantly decreased *TCF/LEF*-dependent transcription of a luciferase reporter gene (*TOPFLASH*) in UMR-106 osteoblastic cells compared with cells treated with vehicle control. Pretreatment of cells with NAC eliminated the effect of EtOH (values are mean ± SD, *n* = 4, *p < .05). (*B*) Representative UMR-106 osteoblastic cell with full-length *β-catenin* nuclear translocated together with nuclear-targeted red fluorescent protein (nRFP) exposed to EtOH 50 mM for 6 hours or pretreated with NAC for 30 minutes and then exposed to EtOH 50 mM for 6 hours.

### Oxidative stress–mediated suppressive effects of EtOH on osteobast differentiation are associated with reduced β-catenin and phosphorylated GSK-3β in vitro

EtOH-inhibited Wnt/β-catenin-mediated transcription led us to hypothesize that EtOH may affect mesenchymal stem cell commitment and osteoblast differentiation. To test this hypothesis, we first treated neonatal rat calvarial osteoblastic cells isolated from untreated 4-day-old rat pups with EtOH, NAC, and their combination for 5 days in the presence or absence of osteogenic medium. In the presence of osteogenic medium, EtOH suppressed alkaline phosphatase (ALP) activity (5*A*). Real-time PCR consistently showed that *ALP* and *osteocalcin* gene expression were downregulated by EtOH (5*C*). Similarly, using primary rat bone marrow cells cultured in the presence of osteogenic medium, EtOH inhibition of nodule formation and bone mineralization was clearly observed (5*B*). These results indicate that EtOH exerts its biologic function by inhibiting or slowing down osteoblast lineage differentiation. To further test whether Wnt signaling is directly involved in EtOH-induced inhibitory effects on osteoblast lineage differentiation, we treated the bone marrow stromal cell line ST2 cells with soluble Wnt3a in the presence of either EtOH, NAC, or their combination. As expected, Wnt3a stimulated osteoblast differentiation. However, this Wnt3a-induced osteoblast differentiation, when evaluated by ALP activity staining, was inhibited or delayed by exposing cells to EtOH (6*A*). Western blotting showed dramatic downregulation of total *β-catenin* levels by EtOH (6*B*). Consistent with previous in vivo data, EtOH-induced degradation of *β-catenin* in ST2 cells was associated with activation of *GSK3β* (6*B*). In addition, we found that protein carbonyl, a marker of cumulative oxidative stress, was elevated (*p* < .05) in EtOH-treated cells (6*C*). NAC blocked all the preceding effects of EtOH without affecting control cells.

**Fig. 5:**
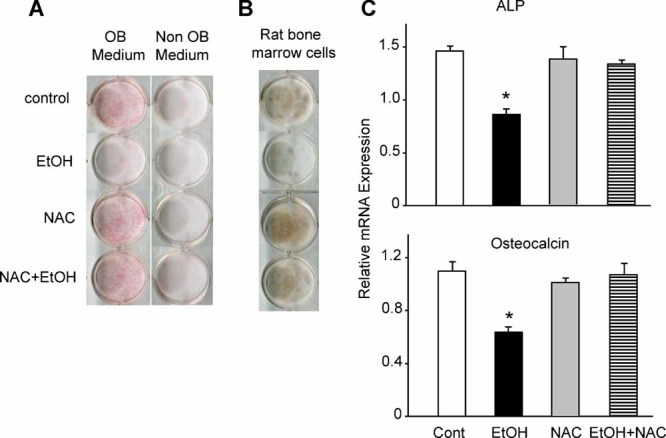
EtOH inhibits osteoblast differentiation. (*A*) Primary osteoblastic neonatal calvarial cells were cultured in the presence or absence of osteogenic medium. Cells were treated with vehicle control, EtOH 50 mM, NAC 1 mM, and NAC + EtOH for 5 days. Alkaline phophatase staining was performed. (*C*) *ALP* and *osteocalcin* mRNA expression measured using real-time PCR. (*B*) Aspirated bone marrow cells from a control rat femur cultured in the presence of osteogenic medium and treated with vehicle control, EtOH 50 mM, NAC 1 mM, and NAC plus EtOH for 25 days followed by von Kossa staining. Bars are expressed as mean ± SD. *p < .05 versus TEN control group by ANOVA followed by Student-Newman-Keuls post hoc analysis for multiple pairwise comparisons.

**Fig. 6:**
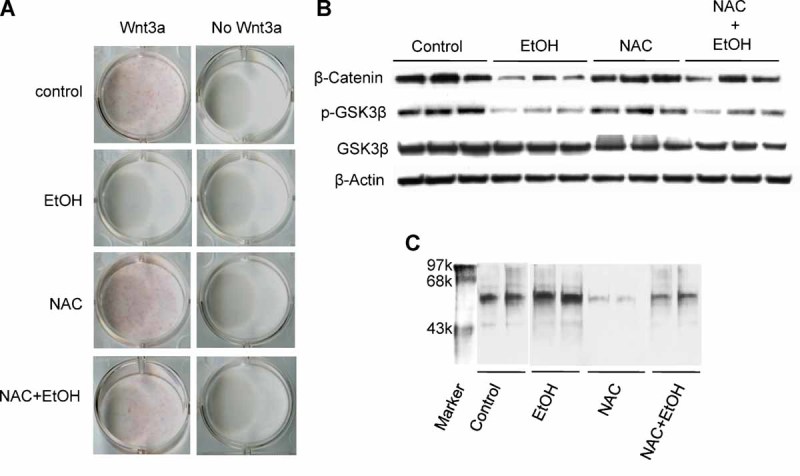
Oxidative stress–dependent suppression of Wnt-driven osteoblastogenesis by EtOH. (*A*) ST2 cells were treated with vehicle control, EtOH 50 mM, NAC 1 mM, or their combination for 7 days in the presence or absence of soluble Wnt3a. Pink color represents alkaline phophatase staining. (*B*) Western blots of β-catenin, GSK-3β, phosphorylated GSK-3β, and β-actin. (*C*) Immunoblot of total protein carbonylation as a measure of oxidative stress.

### EtOH promotes adipogenesis through accumulated oxidative stress

Since osteoblasts and bone marrow adipocytes are believed to originate from the same population of mesenchymal stem cells, and based on our earlier study where we observed significantly increased adiposity in bone marrow of EtOH infused rats,(15) we hypothesized that EtOH not only suppresses osteoblast differentiation but also reciprocally promotes adipogenesis. To directly test this hypothesis, we used a previously established method to induce C3H10T1/2 mesenchymal stromal cells to differentiate into adipocytes. One group of cells was pretreated with EtOH for 2 days prior to exposure to adipogenic medium, and EtOH exposure continued until the end of the experiment. EtOH significantly increased triglyceride-containing adipocytes (7*A*), and real-time PCR revealed dramatic *aP2* and *PPARγ* up-regulation after EtOH treatment (7*B*). NAC blocked EtOH stimulation of adipogenesis and decreased EtOH-induced *aP2* and *PPARγ* gene expression to nearly control levels (7*B*). These data suggest that EtOH is able to enhance adipogenesis, and this process may be mediated through accumulated oxidative stress.

**Fig. 7:**
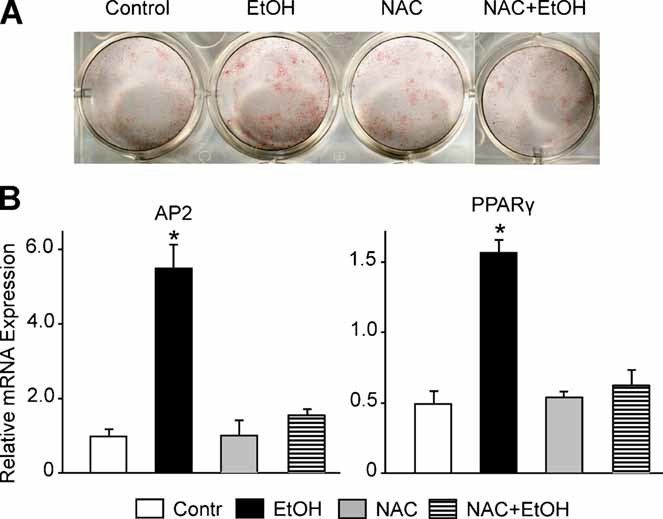
Oxidative stress–mediated promotion of adipogenesis by EtOH. (*A*) C3H cells were cultured with vehicle control, EtOH 50 mM, NAC 1 mM, or NAC plus EtOH for 2 days, after which confluent cells were continued on EtOH and/or NAC treatment supplemented with methyl-isobutylxanthine (0.5 mM), dexamethasone (0.25 µm), and insulin (10 µg/mL) for 2 days to induce adipogenesis. Oil red staining of triglycerides was performed. Alternatively, (*B*) cell RNA was extracted and real-time PCR of *aP2* and *PPARγ* mRNA gene expression was measured. Bars are mean ± SD in triplicate. *p < .05 versus TEN control group by ANOVA followed by Student-Newman-Keuls post hoc analysis for multiple pairwise comparisons.

## Discussion

Chronic alcohol consumption causes damage to a variety of tissues and is a major risk factor for the development of osteoporosis. Most women stop drinking alcohol during pregnancy and lactation because of their awareness of the harmful effects of alcohol on their offspring. However, in many cases they return to drinking immediately after lactation ends. Studies on the toxic effects of alcohol on bone after lactation are extremely limited. In general, it has been recognized that the toxic effect of EtOH on the skeleton results from both direct effects and indirect influence on bone cells as the result of endocrine disruption.(22) However, it is not well understood how alcohol directly influences a bone cell and by what underlying mechanisms. In contrast to EtOH infusion in cycling female rats, where we have reported previously that EtOH primarily increases bone resorption,(14) EtOH appears to induce a dramatic and rapid reduction in bone formation in postlactational female rats at concentrations that mimic the blood alcohol concentrations observed in human alcoholics.(28) Decreased BMD in alcohol-infused animals was associated with reduced serum bone-formation markers and remarkable downregulation of Wnt signaling components. This is consistent with our previous observation that bone-formation parameters and rate were significantly decreased in EtOH-infused postlactational female rats.(15)NAC, a dietary antioxidant precursor of glutathione, blocked the effects of alcohol on bone, consistent with the emerging view that oxidative stress plays a critical role in alcohol-induced bone pathology.

Bone formation is highly associated with osteoblast differentiation and activity and tightly coupled with appropriate functional osteoclast numbers. Under normal conditions, bone resorption is much faster than formation. An area of bone can be resorbed in 2 to 3 weeks, but it takes at least 3 months to rebuild it.(29) During lactation, additional maternal bone resorption is required to provide sufficient mineral to offspring for bone development. After lactation ends, the bone resorbed during lactation is rebuilt in a short period of time. However, the molecular mechanisms underlying the dramatic rebuilding of bone during this period are unknown. This postnatal rebuilding of excessively resorbed bone may be considered as microskeletal tissue regeneration. This process can be disrupted by numerous systemic or local factors, including oxidative stress.(30) EtOH metabolism in osteoblasts that express high levels of alcohol dehydrogenase(22) results in accumulation of reactive oxygen species (ROS).(17) NAC may exert its protective effects on bone through several different mechanisms. It has been reported to increase tissue glutathione concentrations to prevent bone loss in ovariectomized mice(31) and also suppresses expression of NADPH oxidase enzymes (Nox) to reduce ROS generation in osteoblasts.(17) Increased Nox activity in osteoblasts after EtOH exposure depends on several intracellular signals that are sensitive to ROS, including NF-κB, JNK, and ERK MAP kinase(32) and perhaps also the Wnt signaling components described herein. However, the relationship between EtOH and NAC on reciprocal regulation of cytokines and MAP kinases in osteoblasts and osteoblast precursors to determine final cell fate requires further research.

We previously hypothesized that chronic EtOH treatment of female rats in vivo causes accumulation of ROS and may interfere with osteoblast differentiation.(17) This is supported by evidence that EtOH decreases colony-forming units–osteoblast (CFU-OB) formation ex-vivo by cells isolated from bone marrow.(33) The remaining question is whether EtOH can directly inhibit osteoblast differentiation in vitro. To test this hypothesis, we have used several different established cell lines as well as primary bone marrow stromal cells and calvarial cells for in vitro cell culture studies. Our results indicate that the effect of EtOH on osteoblastogenesis resulted from accumulation of oxidative stress and downstream inhibition of Wnt signaling cascades. It appears that EtOH has direct actions in mesenchymal stem cells to block *β-catenin* nuclear translocation and Wnt-induced *TCF/LEF*-dependent transcription at least in part through activation of *GSK3β*. Osteoblasts and adipocytes originate from the same stem cells,(34) and considerable evidence exists to support the idea that a shift in bone marrow stromal cell differentiation to favor the adipocyte lineage over the osteoblast lineage can contribute directly to imbalances in bone formation and ultimately lead to bone loss. In human and experimental animals with chronic alcohol intake, it has been suggested that impaired bone formation is associated with an accumulation of adipose tissue inside the marrow cavity.(35) This relationship suggests a reciprocal regulation of osteoblastogenesis and adipogenesis by alcohol, either directly or indirectly. Although our previously published data demonstrate that EtOH treatment of rats in vivo resulted in increased adiposity in bone marrow,(15) the underlying mechanism of such EtOH-induced adiposity in bone marrow was not clear. In this report we provided further evidence that EtOH, besides its ability to suppress osteoblastogenesis, is also able to facilitate adipogenesis in stem cells when induced in vitro using methyl-isobutylxanthine, dexamethasone, and insulin.(36) This is not only consistent with our earlier hypothesis that alcohol-, aging-, and perhaps sex steroid deficiency–induced bone loss may share common mechanisms but also agrees with the current view that the majority of conditions associated with bone loss coincide with increased marrow adiposity.(37,38) Adipocyte formation is likely a multistep process involving many cellular intermediates typically having two major steps, determination and terminal differentiation. Although we do not know exactly how EtOH influences adipogenesis, it may enhance early adipocyte commitment. Our data demonstrate that EtOH suppresses Wnt signaling and activates *PPARγ* in bone marrow stromal cells. Suppression of *β-catenin* by EtOH also was found in another recent animal study(11) and in a human bone marrow stromal cell study.(41)

It needs to be noted that EtOH also increases tumor necrosis factor α (TNFα) expression in bone marrow.(15) TNFα has been shown in an vitro study to induce early aspects of adipocyte differentiation. However, it prevents the terminal differentiation to adipocytes probably by stimulating canonical Wnt signaling.(39) Based on these data, EtOH-induced TNFα might be expected to stimulate β-catenin signaling and inhibit adipogenesis in bone marrow. However, EtOH-induced increases in bone marrow adiposity have been observed both by us and by other investigators.(15,35,40) We speculate that there are other powerful intracellular signals, such as the p53 pathway,(18) that are also activated by EtOH and which may overwhelm any TNFα signal resulting in β-catenin degradation. Interestingly, NAC blocked the effect of EtOH on promotion of differentiation from mesenchymal stromal cells toward adipocytes, indicating that EtOH-induced oxidative stress may drive cell lineage commitment. On the other hand, our data also indicated that Wnt signaling in osteoblasts may be essential for mesenchymal progenitor cells to differentiate away from a default adipogenic fate into an osteoblastic lineage. Although ST2 and C3H cells are similar mouse-originated mesenchymal stromal cells, it needs to be mentioned that in our in vitro adipogenesis experiments we found that C3H cells were easier to induce adipogenesis in than ST2 cells. Further studies are required to determine the reasons for this, but this is beyond the scope of this report.

In summary, downregulation of canonical Wnt/β-catenin signaling is correlated with suppression of anabolic bone rebuilding by chronic alcohol in postlactational female rats. Alcohol blocked β-catenin nuclear translocation and targeted gene transcription in vitro. Our data indicate that alcohol not only inhibits mature osteoblast activity but also influences the balance between osteoblast and adipocyte differentiation and mesenchymal stem cell commitment in bone marrow. NAC was able to inhibit alcohol-induced adipogenesis, further suggesting that alcohol triggers activation of specific pathophysiologic signaling pathways in bone owing to oxidative stress. Treatment with NAC or other dietary antioxidants may prove valuable in protecting against alcohol-induced bone loss.
