# Supplementary material for: A Role for Ethanol-Induced Oxidative Stress in Controlling Lineage Commitment of Mesenchymal Stromal Cells Through Inhibition of Wnt/β-Catenin Signaling
Source: J Bone Miner Res. 2009 Dec 21;25(5):1117–27. doi: 10.1002/jbmr.7 (PMC3153370; doi:10.1002/jbmr.7)
Supplement: Supplementary file 1 [file jbmr0025-1117-SD1.doc]

Supplemental Table 1. Real-Time Reverse-Transcription Polymerase Chain Reaction (RT-PCR) Primer Sequences.

| Gene | Forward Primer | Reverse Primer |
| --- | --- | --- |
| Rat |  |  |
| ALP | TGAATCGGAACAACCTGACTGA | TTCCACTAGCAAGAAGAAGCCTTT |
| Osteocalcin | [AAGCCCAGCGACTCTGAGTCT](https://www.idtdna.com/OrderStatus/SpecSheet.aspx?OrderNum=4432896&MfgID=32825295&SearchDays=&SearchNum=&SearchPO=&SearchRef=) | [GCTCCAAGTCCATTGTTGAGGTA](https://www.idtdna.com/OrderStatus/SpecSheet.aspx?OrderNum=4432896&MfgID=32828262&SearchDays=&SearchNum=&SearchPO=&SearchRef=) |
| Wnt5a | AGCGGCCATGAGGTTGAAC | CCGGACTTGGGTCGATGTAG |
| LRP6 | GAATGAATGTGCCTCCAGCAA | TTGTCAGCATTCAGGGAGTAGTG |
| Fzd4 | GTACAGCCGCTCAGCTAAGGA | TCAATCAGGAAGGTCAGCACAGT |
| DKK1 | CCGGTTCTTGGTCGTGCTT | TGATCGCGTTGGAATTGATG |
| Cyclophilin | AGCATACAGGTCCTGGCATCTT | GCCATCCAGCCACTCAGTCT |
| GAPDH | [TGAGGTGACCGCATCTTCTTG](https://www.idtdna.com/OrderStatus/SpecSheet.aspx?OrderNum=4432896&MfgID=32825294&SearchDays=&SearchNum=&SearchPO=&SearchRef=) | [TGGTAACCAGGCGTCCGATA](https://www.idtdna.com/OrderStatus/SpecSheet.aspx?OrderNum=4432896&MfgID=32826628&SearchDays=&SearchNum=&SearchPO=&SearchRef=) |
| Mouse |  |  |
| ALP | TAACCGCTACCCGGATCCTA | TGTCTTGGACAGAGCCATGTG |
| Osteocalcin | TTGTGCTGGAGTGGTCTCTATGAC | CACCCTCTTCCCACACTGTACA |
| aP2 | [CAAAATGTGTGATGCCTTTGTG](https://www.idtdna.com/OrderStatus/SpecSheet.aspx?OrderNum=5110341&MfgID=82012624&SearchDays=&SearchNum=&SearchPO=&SearchRef=) | [CTCTTCCTTTGGCTCATGCC](https://www.idtdna.com/OrderStatus/SpecSheet.aspx?OrderNum=5110341&MfgID=82012259&SearchDays=&SearchNum=&SearchPO=&SearchRef=) |
| PPARγ | [CACAATGCCATCAGGTTTGG](https://www.idtdna.com/OrderStatus/SpecSheet.aspx?OrderNum=5110341&MfgID=82012258&SearchDays=&SearchNum=&SearchPO=&SearchRef=) | [GCTGGTCGATATCACTGGAGATC](https://www.idtdna.com/OrderStatus/SpecSheet.aspx?OrderNum=5110341&MfgID=82012623&SearchDays=&SearchNum=&SearchPO=&SearchRef=) |
| Cyclophylin | AAGGTGGAGAGCACCAAGACA | GCAATGGCGAAGGGTTTCT |
| GAPDH | GTATGACTCCACTCACGGCAAA | GGTCTCGCTCCTGGAAGATG |
