# Supplementary material for: A Role for Ethanol-Induced Oxidative Stress in Controlling Lineage Commitment of Mesenchymal Stromal Cells Through Inhibition of Wnt/β-Catenin Signaling
Source: J Bone Miner Res. 2009 Dec 21;25(5):1117–27. doi: 10.1002/jbmr.7 (PMC3153370; doi:10.1002/jbmr.7)
Supplement: Supplementary file 2 [file jbmr0025-1117-SD2.doc]

Supplemental Table 2. Wnt signaling gene list

| Wnt Signaling Genes | | | | | | |
| --- | --- | --- | --- | --- | --- | --- |
|  | EtOH vs TEN | | EtOH+NAC vs EtOH | | NAC vs TEN | |
|  | Fold Up- or Down- Regulation | P value | Fold Up- or Down- Regulation | P value | Fold Up- or Down- Regulation | P value |
| Apc | -2.99 | 0.4335 | 8.24 | 0.0166 | 4.25 | 0.0554 |
| Apc2_predicted | -7.25 | 0.0210 | 3.54 | 0.1744 | -1.53 | 0.7540 |
| Axin1 | -6.98 | 0.0301 | 13.08 | 0.0464 | 8.89 | 0.0317 |
| Axin2 | -3.75 | 0.3619 | 6.69 | 0.0354 | 5.46 | 0.1304 |
| Bcl9_predicted | -2.87 | 0.2311 | 4.41 | 0.1157 | 2.32 | 0.3415 |
| Btrc | -1.04 | 0.9731 | 6.72 | 0.0154 | 6.11 | 0.0143 |
| Ctnnb1 | -75.70 | 0.0115 | 81.51 | 0.0008 | -1.14 | 0.9565 |
| Ccnd1 | -22.14 | 0.0443 | 17.70 | 0.0013 | 4.23 | 0.1257 |
| Ccnd2 | -1.54 | 0.7297 | 9.98 | 0.0110 | 8.39 | 0.0153 |
| Ccnd3 | -1.11 | 0.9439 | 31.03 | 0.0138 | 20.24 | 0.0173 |
| Csnk1a1 | -2.56 | 0.4701 | 5.07 | 0.2399 | 2.81 | 0.1382 |
| Csnk1d | -1.18 | 0.8966 | 10.84 | 0.0118 | 9.98 | 0.0288 |
| Csnk2a1 | -3.31 | 0.0388 | 9.27 | 0.0139 | 3.82 | 0.0882 |
| Csnk2b | -3.80 | 0.0348 | 6.63 | 0.2034 | 2.79 | 0.1587 |
| Daam1_predicted | 2.73 | 0.3335 | 12.75 | 0.1815 | 9.08 | 0.0262 |
| Dixdc1_predicted | -2.66 | 0.4418 | 2.88 | 0.1760 | 1.85 | 0.6022 |
| Dkk1_predicted | 3.14 | 0.0482 | -17.08 | 0.0105 | -6.44 | 0.0259 |
| Dkk3 | 1.85 | 0.5264 | 5.97 | 0.1444 | 7.44 | 0.0335 |
| Dkk4_predicted | -1.18 | 0.9426 | 1.63 | 0.5145 | -1.34 | 0.8290 |
| Dvl1 | -1.70 | 0.6260 | 15.62 | 0.0522 | 8.35 | 0.0472 |
| Dvl2 | 1.40 | 0.8583 | 8.26 | 0.0379 | 2.69 | 0.4859 |
| Dvl3_predicted | -1.86 | 0.5445 | 4.86 | 0.1655 | 4.05 | 0.0764 |
| Ep300 | 1.12 | 0.9446 | 16.17 | 0.0140 | 21.79 | 0.0054 |
| Fbxw11_predicted | -1.73 | 0.6790 | 9.06 | 0.1287 | 6.33 | 0.0698 |
| Fbxw2_predicted | -1.49 | 0.8049 | 13.02 | 0.0151 | 11.18 | 0.0219 |
| Fgf4 | -26.83 | 0.0163 | -1.78 | 0.3434 | -9.48 | 0.1622 |
| Frzb | -7.67 | 0.0123 | 2.06 | 0.3433 | -1.55 | 0.6663 |
| Fzd1 | -6.64 | 0.0261 | 5.35 | 0.0894 | 3.09 | 0.1151 |
| Fzd2 | -6.52 | 0.0202 | 2.68 | 0.1777 | -1.22 | 0.8727 |
| Fzd3 | -12.14 | 0.0150 | 1.65 | 0.6322 | -1.09 | 0.9050 |
| Fzd4 | -3.19 | 0.3131 | 10.72 | 0.0301 | 2.85 | 0.2710 |
| Fzd5 | -3.91 | 0.2315 | 6.42 | 0.0499 | 1.21 | 0.8314 |
| Fzd6 | -21.89 | 0.0418 | 5.04 | 0.0651 | 1.66 | 0.4561 |
| Fzd7_predicted | -21.84 | 0.0216 | 5.07 | 0.0437 | 1.15 | 0.9226 |
| Fzd9 | -44.70 | 0.0150 | -1.57 | 0.4518 | -5.96 | 0.3073 |
| Gsk3a | 7.28 | 0.1268 | 14.34 | 0.1993 | 12.06 | 0.0424 |
| Gsk3b | -3.02 | 0.0515 | 12.54 | 0.0176 | 4.72 | 0.0825 |
| Jun | 2.97 | 0.1224 | 13.38 | 0.1342 | 6.19 | 0.0418 |
| Kremen1 | 1.54 | 0.6893 | 5.38 | 0.2327 | 1.84 | 0.3741 |
| Lef1 | 2.78 | 0.2828 | 5.29 | 0.2412 | 4.41 | 0.0413 |
| Lrp5_predicted | -6.37 | 0.0330 | 8.22 | 0.0213 | 2.64 | 0.3826 |
| Lrp6_predicted | -2.03 | 0.6400 | 10.14 | 0.0159 | 7.31 | 0.0532 |
| Mitf | -1.81 | 0.6275 | 8.05 | 0.1259 | 6.34 | 0.0387 |
| Myc | -2.23 | 0.5869 | 19.46 | 0.0346 | 10.55 | 0.0250 |
| Nkd1_predicted | -1.42 | 0.7691 | 8.59 | 0.0245 | 4.86 | 0.2337 |
| Nkd2_predicted | -1.48 | 0.6687 | 5.04 | 0.0591 | 3.96 | 0.1852 |
| RGD1561440_predicted | -1.43 | 0.7675 | 13.08 | 0.0468 | 9.77 | 0.0146 |
| Pitx2 | -5.50 | 0.0318 | -1.83 | 0.5393 | -5.48 | 0.2721 |
| RGD1564947_predicted | -2.79 | 0.3215 | 4.22 | 0.1584 | 2.01 | 0.3522 |
| Ppp2ca | -1.90 | 0.7152 | 17.41 | 0.0149 | 12.34 | 0.0150 |
| Ppp2r1a | -4.40 | 0.0415 | 14.48 | 0.0177 | 4.97 | 0.0789 |
| RGD1308535_predicted | 1.66 | 0.7596 | 9.14 | 0.0418 | 5.63 | 0.2923 |
| Rhoa | -1.97 | 0.6999 | 12.69 | 0.0190 | 9.55 | 0.0177 |
| Senp2 | -1.84 | 0.6880 | 7.53 | 0.2245 | 5.08 | 0.0356 |
| Sfrp1 | -8.47 | 0.0175 | -1.47 | 0.5264 | -9.48 | 0.1622 |
| Sfrp2 | -2.73 | 0.3281 | 7.12 | 0.0955 | 4.12 | 0.0957 |
| Sfrp4 | -2.59 | 0.3289 | 7.46 | 0.0554 | 1.64 | 0.4867 |
| Sfrp5_predicted | -2.49 | 0.5669 | 1.98 | 0.4139 | -1.40 | 0.8361 |
| Tcf3_predicted | -2.40 | 0.0557 | 18.41 | 0.0096 | 3.55 | 0.3635 |
| Tcf4 | -1.39 | 0.7882 | 4.83 | 0.2425 | 5.51 | 0.0242 |
| Tcf7_predicted | -2.23 | 0.0414 | 7.14 | 0.0386 | 3.67 | 0.0932 |
| Tcfe2a | -1.38 | 0.8273 | 20.14 | 0.0287 | 16.02 | 0.0409 |
| Tle1_predicted | -2.29 | 0.5976 | 2.42 | 0.2797 | 1.05 | 0.9463 |
| Tle2 | -5.34 | 0.0149 | 2.05 | 0.4115 | -1.12 | 0.8581 |
| Wif1 | -4.48 | 0.3134 | 1.61 | 0.4094 | -1.85 | 0.6497 |
| Wisp1 | -6.27 | 0.1406 | 3.42 | 0.1772 | 1.01 | 0.9888 |
| Wnt1 | -17.86 | 0.0110 | -1.78 | 0.3434 | -6.86 | 0.1949 |
| Wnt10a_predicted | -6.33 | 0.0278 | 3.29 | 0.2421 | -2.03 | 0.6686 |
| Wnt10b_predicted | -3.28 | 0.4062 | -1.28 | 0.8169 | -1.85 | 0.6618 |
| Wnt11 | -10.33 | 0.0222 | 1.92 | 0.3261 | -3.79 | 0.4242 |
| Wnt2 | -5.29 | 0.0178 | 6.33 | 0.0499 | 2.51 | 0.2881 |
| Wnt2b | -7.09 | 0.0149 | 4.17 | 0.0455 | 1.42 | 0.6727 |
| Wnt3 | -7.87 | 0.0191 | -1.52 | 0.4843 | -8.42 | 0.1912 |
| Wnt3a_predicted | -11.65 | 0.0221 | -1.53 | 0.5560 | -5.19 | 0.2940 |
| Wnt4 | -10.24 | 0.0131 | 6.21 | 0.0241 | 2.23 | 0.4541 |
| Wnt5a | -1.63 | 0.7857 | 2.64 | 0.4518 | 7.27 | 0.1825 |
| Wnt5b | -2.34 | 0.4110 | 5.19 | 0.0114 | 3.30 | 0.3313 |
| Wnt6_predicted | -14.01 | 0.0227 | 3.46 | 0.1498 | -1.43 | 0.8116 |
| Wnt7a | -9.66 | 0.0192 | -1.77 | 0.3649 | -11.89 | 0.1610 |
| Wnt7b | -3.34 | 0.4475 | -1.78 | 0.3434 | -9.48 | 0.1622 |
| Wnt8a_predicted | -14.05 | 0.0128 | -1.78 | 0.3434 | -9.48 | 0.1622 |
| Wnt8b_predicted | -15.91 | 0.0162 | -1.44 | 0.5471 | -6.95 | 0.2224 |
| Wnt9a_predicted | -12.23 | 0.0104 | -1.13 | 0.8468 | -6.20 | 0.2296 |
| Wnt9b_predicted | -7.87 | 0.0191 | -1.16 | 0.7898 | -8.52 | 0.1799 |
| Rplp1 | -2.27 | 0.1382 | 2.63 | 0.0700 | -3.20 | 0.2387 |
| Hprt | 6.53 | 0.0543 | -6.52 | 0.0488 | 1.92 | 0.2396 |
| Rpl13a | -2.87 | 0.1318 | 2.48 | 0.1790 | 1.67 | 0.2802 |
| Ldha | -2.99 | 0.4335 | -6.37 | 0.0756 | 58.58 | 0.0072 |
| Actb | -7.25 | 0.0210 | 3.31 | 0.1602 | 21.24 | 0.0200 |

The table represents real-time PCR array data. RNA isolated from bone marrow cells with three samples per group. A list of total 89 genes identified as affiliated with the Wnt signaling pathway by the PANTHER classification system that were also identified as differentially expressed (p<0.05) in bone marrow between TEN control and EtOH infused, EtOH infused and NAC plus EtOH infused, TEN control and NAC treated rat groups. Data were normalized with housekeeping gene GAPDH and statistical comparisons analyzed based on a ∆∆Ct method with excel-based PCR array data template form provided by the manufacturer. Red labeled 36 genes were found having 3 fold changes and p value less than 0.05 between TEN and EtOH groups. Among those 36 genes, there are 12 genes labeled blue having 3 fold changes and p value less than 0.05 between EtOH and EtOH plus NAC, two genes labeled green have 3 fold changes and p value less than 0.05 between TEN and NAC. Minus symbol =down-regulated. The individual full gene name can be found in reference 11.
